# Isolation, Purification, and Antimicrobial Characterization of Cannabidiolic Acid and Cannabidiol from *Cannabis sativa* L.

**DOI:** 10.3390/biom10060900

**Published:** 2020-06-12

**Authors:** Laura Daniela Martinenghi, Rie Jønsson, Torben Lund, Håvard Jenssen

**Affiliations:** Department of Science and Environment, Roskilde University, 4000 Roskilde, Denmark; laumarti80@yahoo.es (L.D.M.); riejonsson@gmail.com (R.J.); tlund@ruc.dk (T.L.)

**Keywords:** cannabidiolic acid, cannabidiol, antibacterial, snergy, *Cannabis sativa* L., *Staphyloccoccus*, HPLC, cannabinoids, cannabis

## Abstract

The emergence of multi-drug resistant bacteria such as methicillin-resistant *Staphylococcus aureus* (MRSA) causes a major threat to public health due to its limited therapeutic options. There is an urgent need for the development of new effective antimicrobial agents and alternative strategies that are effective against resistant bacteria. The parallel legalization of cannabis and its products has fueled research into its many therapeutic avenues in many countries around the world. This study aimed at the development of a reliable method for the extraction, purification, characterization, and quantification of cannabidiolic acid (CBDA) and its decarboxylated form cannabidiol (CBD) present in the fiber type *Cannabis sativa* L. The two compounds were extracted by ethanol, purified on a C18 sep-pack column, and the extracts were analyzed by high performance liquid chromatography coupled with ultraviolet (UV)–vis and ESI-MS (electrospray ionization mass spectrometry) detection. The antimicrobial effect of CBDA and CBD was also evaluated. CBD displayed a substantial inhibitory effect on Gram-positive bacteria with minimal inhibitory concentrations ranging from 1 to 2 µg/mL. Time kill analysis and minimal bactericidal concentration revealed potential bactericidal activity of CBDA and CBD. While cannabinoids showed a significant antimicrobial effect on the Gram-positive *S. aureus* and *Staphylococcus epidermidis*, no activity was noticed on Gram-negative *Escherichia coli* and *Pseudomonas aeruginosa*. CBDA presented a two-fold lower antimicrobial activity than its decarboxylated form, suggesting that the antimicrobial pharmacophore of the analyzed cannabinoids falls in the ability for permeabilizing the bacterial cell membrane and acting as a detergent-like agent. A synergy test performed on MRSA with CBD and a range of antibiotics did not indicate a synergetic effect, but noteworthy no antagonist influence either. CBD and CBDA manifested low hemolytic activity on human red blood cells. Likewise, the safety of CBD toward human keratinocyte cells presents no toxicity at a concentration of up to seven-fold higher than the antibacterial minimal inhibitory concentration. Similarly, both CBD and CBDA are well tolerated by mammals, including humans, and conserve a safe value limits for blood-contacting drug development. Overall, CBD exhibited a strong antimicrobial effect against Gram-positive strains and could serve as an alternative drug for tackling MRSA.

## 1. Introduction

**Figure 1:**
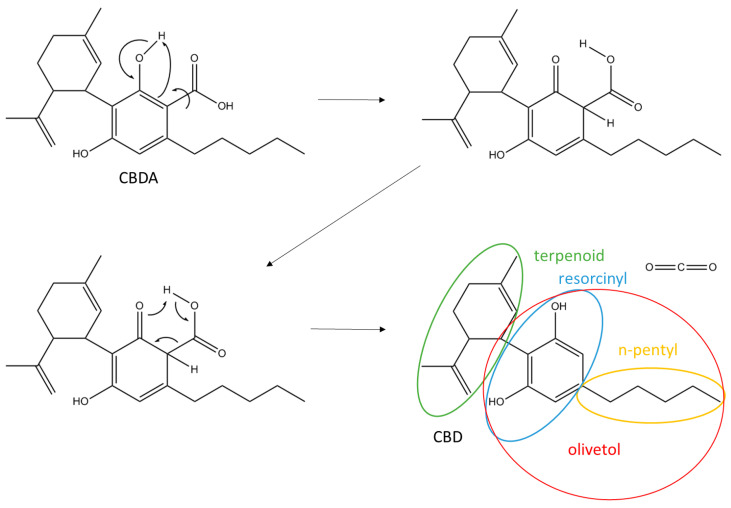
Chemical structure of cannabidiolic acid (CBDA) with a decarboxylation scheme of cannabidiol (CBD).

Cannabis has been a resource for humans for over 5000 years and employed in the food, textile, and medicine industry. It has been associated with a number of pharmacological activities; alleviating chronic pain and muscle spasms, reducing nausea during chemotherapy, enhancing appetite in HIV/AIDS (human immunodeficiency virus/acquired immunodeficiency syndrome) patients, improving sleep, and reducing tics in Tourette syndrome [1]. Moreover, it has been recommended for use in extreme cases of anorexia, arthritis, migraine, and glaucoma [2,3,4]. This medicinal plant encounters three main varieties, *Cannabis sativa* L., *Cannabis indica*, and *Cannabis ruderdalis* [5]. *C. sativa* L. is the most studied in the medical industry due to its ease of cultivation and adaptation to different climates. *Cannabis* strains are also classified in three main phenotypes depending on their expression level of Δ^9^-tetrahydrocannabinolic acid (Δ^9^-THCA). European Union regulations have classified these strains as a drug strain (up to 20% THCA), an intermediate strain (up to 0.5% THCA), and fiber-type (below 0.2% THCA) and different amounts of cannabidiolic acid (CBDA) [6,7]. In addition to THCA and CBDA, more than 550 chemical constituents have been identified from *C. sativa* L., with an increase in new cannabinoids from 70 to 115 in the past years [3,4,8]. Cannabinoids are a type of terpenophenolics, mainly synthesized in glandular trichomes more abundant in female inflorescences and the most important secondary metabolism of *C. sativa* L. [7]. Cannabinoids have been the focus of studies for the pharmaceutical industry, in particular the main psychoactive compound Δ^9^-THC and the non-psychoactive component cannabidiol (CBD) [8]. The primary cannabinoids in the plant exist in acidic form, but due to aging, heat, and ultraviolet (UV) light exposure, both CBDA and THCA de-carboxylate over time to THC and CBD (Figure 1), with a different molecular structure and function. 

*Staphylococcus aureus* is a major human pathogen that causes, among other, bacteremia, endocarditis, osteomyelitis, skin infections, and pneumonia [9]. Bacteria have shown a tremendous ability to adapt under stressed environments; specifically, to evolve resistance to antibiotic treatments and, unfortunately, only one class of new antibacterial agent has been approved in the last 30 years [10,11]. Therefore, actions must urgently be taken to address this problem. Controlling the use and overuse of antibiotics, a better understanding of the mechanism of resistance, and developing a new treatment are the main topics of studies these days [12,13].

Since ancient times, plants have been known as a valuable source of natural products for medicine. Many plants have been utilized because of the great antimicrobial traits, due to secondary metabolites [14,15,16,17,18,19,20]. Even though, in many cases, the underlying mechanism remains somehow unexplained, only understanding how it works will give us the possibility to develop new antimicrobials that fulfill today’s needs for finding an efficient medicine as substitute or for use in combination with antibiotics. This opens a new window towards a multi-drug therapy that is already being practiced among some diseases that have a multi-causal etiology and complex pathophysiology, such as AIDS, and to some extent against multi-resistant bacterial infections [21,22,23,24].

In pursuit of a further investigation of the action mechanism of the cannabinoids of *C. sativa* L. against bacteria, the purpose of this study is to have an overview of the antimicrobial effect of the isolated CBDA from the crude extract of *C. sativa* L. from Denmark, its decarboxylated form CBD, comparing them with the effects of both bacteriostatic and bactericidal antibiotics, such as tobramycin, clindamycin, ofloxacin, and meropenem on *S. aureus*, methicillin-resistant *Staphylococcus epidermidis*, *S. epidermidis*, *Escherichia coli,* and *Pseudomonas aeruginosa*. Furthermore, the purpose of this study is to evaluate the antimicrobial effect of CBD and CBDA on methicillin-resistant *S. aureus* (USA300) and parallelly test the possible synergy between these compounds with classical antibiotics. This investigation not only aims to gain knowledge of the possible synergetic effects of these combinations, but to moreover compare them with single constituents.

## 2. Material and Methods

The strains of fiber-type *Cannabis sativa* L. was provided by Canna Therapeutics (Stenløse, Denmark) and Møllerup Gods (Rønde, Denmark). The inflorescences (clusters of flowers) were harvested and used for isolation of CBDA and CBD (Figure 1). Separate stock solutions of CBDA and CBD were prepared in ethyl acetate and methanol, respectively, at a concentration of 100, 200, 500, and 750 µg/mL and stored at −20 °C. 

### 2.1. Isolation of Cannabinoids

After harvest, the plant material was air-dried in the dark under constant temperature and humidity conditions until use. Twelve grams of cannabis were finely grinded and added to 200 mL of ethanol, extractions were made in triplicates. The mixture was macerated for 4 h under continuous stirring at room temperature. The extracts were divided into two, filtered over a Whatman (Maidstone, UK) filter paper no. 1 under reduced pressure, and further passed through a cellulose acetate syringe filter (0.2 µm pore size) before the solvent was evaporated under reduced pressure at 40 °C. Each residue was weighed, and the percentage yield was determined. The dry extract was resolubilized in 5:1 methanol:water to a final volume of 50 mL and passed through a chromatography setup with a large 10 g bed mass RP-C18 sep-pack column from Waters (Taastrup, Denmark), (UNSPSC code 41115712). Fractions were eluted (20 mL) with a step gradient, starting at 10:90 (methanol:water) and increasing the organic solvent concentration by 10% steps until 100% methanol was reached. After HPLC analysis, all fractions containing cannabinoids were pooled together. The sample from Møllerup Gods was further purified by gravity column chromatography on silica gel using a petroleum ether-EtOAc gradient (from 8:2 to 5:5) to afford a set of 20 fractions and monitored by thin layer chromatography on Merck (Søborg, Denmark) TLC silica gel 60 F254 (0.25 mm) and analyzed by HPLC. Fractions that contained CBDA were pooled together. After removal of the solvent under reduced pressure, the remaining water was lyophilized in a bench-top freeze dryer over two days. The powder was stored at −20 °C until use. The sample from Canna Therapeutics (Stenløse, Denmark) was re-suspended in 2 mL dimethyl sulfoxide and stored at 4 °C until further use.

### 2.2. High Performance Liquid Chromatography

Analyses were carried out using a Thermo Fisher Scientific (Roskilde, Denmark) Dionex UltiMate 3000 series UHPLC (ultra-high-performance liquid chromatography) system, consisting of a 1000 bar binary UHPLC pump and an XRS autosampler. Full spectra were recorded in the range of 200–400 nm using a UV 6000LP Photodiode array UV/vis detector and MS detector Finnigan LQT (XL) equipped with Heated Electro Spray Ion source (HESI II) Thermo Fisher Scientific (Roskilde, Denmark). Chromatographic separation was achieved using a Phenomenex (Værløse, Denmark) C18 column, type Kinetex, 2.6 µm, 2.1 × 100 mm. Equipment control, data acquisition, and integration were performed with Xcalibur 2.2 SP1, Thermo Fisher Scientific (Roskilde, Denmark). The mobile phase consisted of a binary A/B gradient. Solvent A consisted of 89% Milli Q water + 1% formic acid + 10% acetonitrile. Solvent B consisted of 0.1% formic acid in 99.9% acetonitrile. The initial setting was 2% B which was linearly increased to 95% acetonitrile after 35 min, this condition was maintained for 5 min, the column was re-equilibrated under initial conditions resulting in a total runtime of 40 min. The flow rate was set at 0.350 mL/min with an increase to 0.500 mL/min at 38 min for 1 min, the column was re-equilibrated under initial conditions for an injection volume of 2 µL, and the detection wavelength was 225–400 nm. The temperature of the column oven was 45 °C. Electro-spray was performed in the positive ionization mode. The conditions were set as follows: a needle voltage of 5.0 kV, heated capillary at 250 °C, a nitrogen flow rate of 22 arb, an auxiliary gas flow of 5 arb, and a sweep gas flow rate of 10 arb. 

### 2.3. Preparation of Standard Solutions

Cannabinoid analytical standards for CBDA and CBD, methanol, ether-petroleum, DMSO, ethanol, ethyl-acetate, formic acid, were all purchased from Sigma-Aldrich (Søborg, Denmark). A stock solution of CBDA (1000 µg/mL) in ethyl-acetate and CBD (1000 µg/mL) in methanol were properly diluted to obtain samples of 100, 200, 500, and 700 µg/mL. The stock solution from the extractions was diluted in methanol to obtain 500, 1000, and 3000 µg/mL. For the bacterial test, dilutions of the dried isolated fractions after C18 columns purification were prepared on Muller-Hinton broth (MHB) media in a range from 1024 µg/mL to 0.03 µg/mL.

### 2.4. Solubility Test

Three milliliters from each CBDA and CBD isolates were taken for the solubility test. Evaporation of the solvent was performed under reduced pressure at room temperature for 45 min. The dried extract from both CBDA and CBD was weighed and re-dissolved in methanol to a final concentration of 1024 µg/mL. In 100 mm round-bottom centrifuge tubes, two-fold series dilutions were performed from 1024 to 0.6 µg/mL using MHB media. Note that neither centrifugation nor heating up to 45 °C improved solubility results.

### 2.5. Bacterial Strains

The cannabis extracts were tested against *S. aureus* (ATCC 25923), methicillin-resistant *S. aureus* (USA300), methicillin-resistant *S. epidermidis* (ATCC 51625), a clinical strain of *S. epidermidis* (CA#71) [25], *E. coli* (ATCC 25922), and *P. aeruginosa* (PA01) (Table 1). All trains were cultivated in Mueller-Hinton broth (MHB; Oxoid) at 37 °C.

### 2.6. Antibiotic Susceptibility Test

The minimum inhibitory concentrations (MICs) and minimum bactericidal concentrations (MBCs) were determined for each isolate in triplicate according to the broth microdilution method according to the guidelines of the National Committee for Clinical Laboratory Standards (CLSI) using standardized methods [26]. Classical antibiotics, i.e., clindamycin, ofloxacin, meropenem, tobramycin, teicoplanin, methicillin, and vancomycin were used as controls. 

### 2.7. Synergy Test

The synergetic effect of CBD, clindamycin, ofloxacin, meropenem, tobramycin, teicoplanin, methicillin, and vancomycin was investigated on *S. aureus* USA300 using the checkboard method [27]. Nine two-fold dilutions from 128 µg/mL to 0.5 µg/mL of the antibiotics and from 16 µg/mL to 0.25 µg/mL of CBD were employed for this test and diluted in MHB. The fractional inhibitory concentration (FIC) was calculated as a ratio of the MIC of the compound in combination and MIC of the compound alone. The FIC_index_ was calculated as the sum of the FIC for the antibiotic (*a*), plus the FIC of CBD (*b*) [27].

$$FIC_{a}=\frac{MIC_{a+b}\;}{MIC_{a}},$$

$$FIC_{b}=\frac{MIC_{b+a}\;}{MIC_{b}},$$

$$FIC_{index}=FIC\;a+FIC\;b.$$

When two drugs are used in combination and at least one shows a decrease in MIC, 1/4 is considered synergy, which is interpreted when the FIC_index_ is ≤0.5. An additive effect is considered when the effect produced by the two agents is comparable to the sum of their separate effects (FIC_index_ between 0.5 and 4). While antagonistic effects are reported for FIC_index_ > 4 [28]. 

### 2.8. Optical Density Measurements

Optical density measurements of bacterial cultures in combination with CBD were performed in a Molecular Devices SpectraMax^®^ i3X (San Jose, CA, USA) in a Nunc Flat Bottom 96-well plate. Absorbance was measured at 600 nm, at 37 °C every 30 min for 22 h. A similar procedure as MIC determination was conducted, plating 50 µL of bacterial culture 1 × 10^6^ CFU (colony forming units)/mL and 50 µL of CBD in concentrations 4 × MIC, 2 × MIC, and MIC. Sterile and positive controls were assigned, and each test was conducted in technical triplicates. Only *S. aureus* ATCC 25923 and USA300 were employed for this test.

### 2.9. In Vitro Hemolysis Essay on Human Red Blood Cells

The ability of CBDA and CBD to induce hemolysis in human erythrocytes was assessed according to Mojsoska et al. [29]. Blood from a randomized anonymous healthy human donor of the type A+, diluted in K2-EDTA-coated Vacutainer tubes, was donated by Blodbanken Roskilde, Sjælland University hospital, Denmark. Hemolytic activity was determined by incubating suspensions of human red blood cells with serial dilutions of 2 × MIC, MIC, and 1/2 × MIC of CBDA and CBD. Negative control included incubation of blood cells with PBS and positive control with 4% SDS. The research covered in the manuscript making use of human red blood cells was approved by the Danish Labor Inspection Authority (Arbejdstilsynet) in respect to “genteknologiske Forskningsprojekter” (11/11/14). Additionally, the blood work has been carried out in accordance with internal safety and ethics regulations approved by the head of Department of Science and Environment at Roskilde University, Denmark. 

### 2.10. Cell Proliferation Assay—MTS

This assay is based on the molecular transformation of the tetrazolium salt, MTS, into formazan that is soluble in the tissue culture medium. Absorbance is directly proportional to the number of living cells in the culture. Human HaCaT keratinocytes cells were seeded into a flat-bottom microtiter plate at a density of 1 × 10^4^ cells/well in a 100 µL volume and allowed to adhere for 24 h at 37 °C in 5% CO_2_. The cells were treated with 10 µL of a two-fold dilution ranging from 64 to 0.5 µg/mL of CBD diluted in water. After 24 h at 37 °C in a 5% CO_2_ incubator with the antibiotic agent, they were treated with 10 µL of MTS reagent and allowed to react for 1.5 h in the same conditions as named above. Negative control was prepared in the absence of CBD. Optical density measurements were performed with Molecular Devices SpectraMax^®^ i3X (San Jose, CA, USA) at 490 nm. All tests were made in triplicates. MTS reagent was stored at −20 °C and equilibrated at room temperature before use.

### 2.11. Time Kill Essay

Time killing studies were conducted for Methicillin-resistant *S. aureus* (USA300) following standard protocols [30]. In brief, 10 mL of a log-phase inoculum of approximately 5 × 10^6^ was inoculated 1:1 with CBD MIC, 2 × MIC, and 4 × MIC. Samples were taken at time 0, 0.5, 1, 2, 4, 6, 12, 20, and 28 h and subsequently ten-fold serial-diluted in 0.9% NaCl. A total of 10 µL of each dilution was plated in triplicates on agar plates and incubated at 37 °C for 24–48 h. Viable bacteria were quantified after 24 and 48 h. Bactericidal activity was defined as 3-log 10 reduction of growth compared with the initial inoculum. Time-kill curves were plotted graphically as log10 CFU/mL versus time.

### 2.12. Statistical Analysis

All experiments were performed in biological and technical triplicates. Statistical analysis was performed using the GraphPadPrism Version 5 software (Graphpad Corporation, San Diego, CA, USA). 

## 3. Results

### 3.1. Isolation and Purification of Cannabidiolic Acid and Cannabidiol

**Figure 2:**
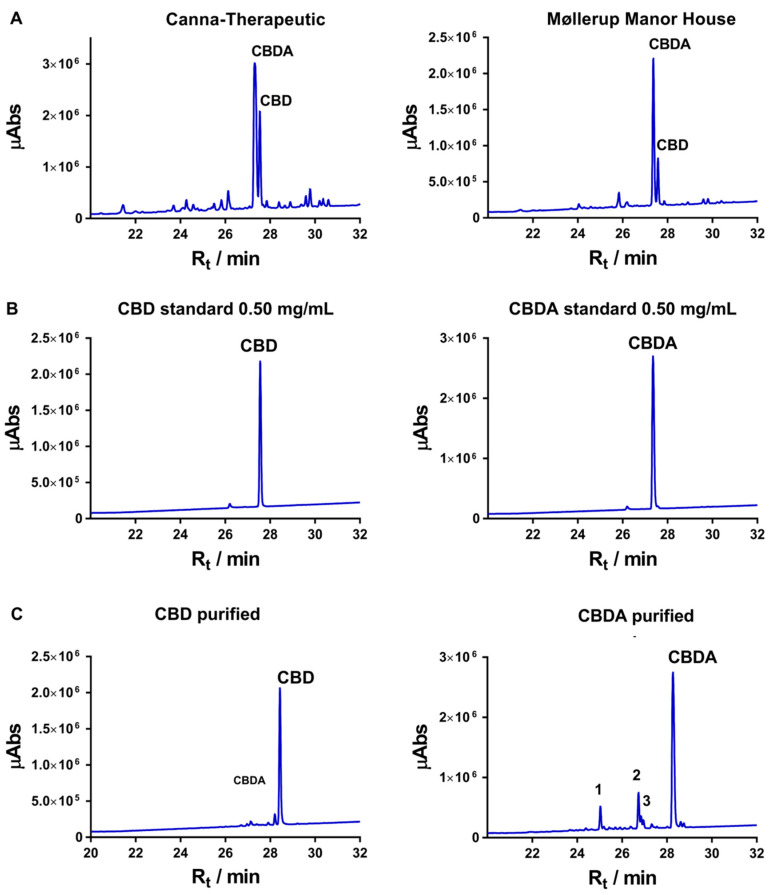
(**A**) HPLC chromatogram of the two cannabis extracts from Canna-Terapeutics and Møllerup Gods. The peaks with retention times (R_t_) of 28.4 and 28.2 min correspond to CBD and CBDA and are present in both the standards (**B**) and the purified samples (data not show). (**C**) Purified CBD and CBDA; the samples are run on a semi-preparative column, thus resulting in different retention times. The LC-UV/vis chromatogram is plotted in λ_max_ (wavelength of maximum absorbance) mode between 225 and 800 nm. Abs: absorbance.

Methanol extraction of 12 g cannabis from Canna Therapeutics and Møllerup Gods resulted in soluble oils amounting to 57 mg and 119 mg, respectively. Fractionation of the oil over a C18 sep-pack column allowed for separation of CBDA and CBD. By pooling all fractions with traces of CBDA and CBD, the yield from Canna Therapeutics was 35.3 mg and the yield from Møllerup Gods was 22.6 mg. The isolation and purification of the different cannabinoids present a challenge when performed under isocratic elution conditions on a large reverse phase C18 solid phase column due to the similar formula and molecular weights of the cannabinoids. The selection of the purest CBDA and CBD fractions were guided by LC-UV/Vis-MS (liquid chromatography-ultraviolet/visible-mass spectrometry) analysis of all fractions. The quantification of the two cannabinoids of interest was achieved by comparison of the peak intensity and integration numbers of CBDA and CBD of the whole extract (Figure 2A). The best peak separation was acquired by the employment of sep-pack C18 columns and gradient elution. Formic acid (0.1%), in the mobile phase, improved the base peak and resolution of the analytes, in particular for the acidic forms. A standard curve of CBDA and CBD was constructed at four calibration levels of 100, 200, 500, and 750 µg/mL. Peak integration ratios were plotted versus actual concentrations. Linearity was determined through the calculation of the coefficient of determination (*R^2^*), which should be greater than 0.98 for a >99% accuracy (Supplementary Figure S1). Both extracts were obtained as a yellowish oil. Drying the inflorescence in an oven at 145 °C for 50 min prior to extraction did not significantly accelerate decarboxylation changing the ratio of CBDA and CBD, though it did impact on the yield. Similarly, heating the extracted cannabinoid solution to 145 °C for 30 min prior to CBDA/CBD purification clearly resulted in the degradation of both CBDA and CBD (data not shown), thus, warranting optimization of both temperature and time should be implemented to control decarboxylation of CBDA in order to increase the yield of CBD.

The processed cannabis extracts preserved the cannabinoids concentration longer when diluted in DMSO rather than in methanol due to a higher boiling point and, consequently, less volatile properties [31]. In either solvent, the CBDA decomposition rate was very low for at least seven days if stored in the dark at 4 °C. The thermal instability of CBDA allows for slow decarboxylation (Figure 1) when stored at 15 °C for 10 days before subsequent HPLC analysis. CBDA presents more instability than CBD since the carboxylic acid group interacts strongly with methanol and water molecules. Expectedly, no decomposition has been experienced in CBD during HPLC analysis. Respecting solubility, CBD, due to the lack of a carboxylic group, obtained full solubility in MHB media at 128 µg/mL. On the other hand, CBDA obtained full solubility in MHB media at 512 µg/mL.

UHPLC, UV/Vis MS analysis of CBDA gave an intense peak with 28.2 min retention time over the C18 column and presented a pseudo molecular ion peak [*M* + H]^+^ at *m/z* 359.2 consistent with a molecular formula of $C_{22}H_{30}O_{4}$. Comparably, CBD gave a less intense peak with 28.4 min retention time and with a molecular ion peak [*M* + H]^+^ at *m/z* 315.3 suggesting the molecular formula $C_{21}H_{30}O_{2}$. Both compound peaks from the analytical standards align with the purified compounds (Figure 2B) and are in accordance with the available literature [31,32]. Integration of the entire chromatograms of the purities of the clean fractions of CBD and CBDA are 89.7 and 84.9%, respectively (Figure 2C, Supplementary Figures S2 and S3).

CBDA and CBD show different UV behavior. CBDA has three absorption maxima (λ_max_), one stronger at 227 nm, a second one at 269 nm, and a third one around 307 nm, while CBD showed first a λ_max_ at 228 nm and an additional UV band at 271 nm. CBDA showed a MS^2^ spectrum with fragmentation of a water molecule, *m/z* 359 → *m/z* 341. The MS^2^ spectrum of CBD was more complicated showing fragmentation of *m/z* 315 → *m/z* 297 (-H_2_O), 259 (-C_4_H_8_), and 193.

### 3.2. Minimum Inhibitory Concentration

The antibacterial activity of purified CBDA and CBD, extracted from *C. sativa* L., was monitored against four Gram-positive organisms, *S. aureus* (ATCC 25923), *S. epidermidis* (ATCC 51625), methicillin-resistant *Staphylococcus aureus* (MRSA) (USA300), and *S. epidermidis* (CA#71), and two Gram-negative organisms, *E. coli* (ATCC 25922) and *P. aeruginosa* (PAO1). Four conventional antibiotics, i.e., clindamycin, ofloxacin, meropenem, and tobramycin, were implemented as a reference. CBD demonstrated a potent activity against Gram-positive bacteria with a minimal inhibitory concentration between 1 and 2 µg/mL followed by CBDA with a two-fold lower activity. Neither CBDA nor CBD presented antibacterial activity against any of the Gram-negative strains at concentrations of 64 µg/mL (Table 1). 

### 3.3. Synergy Test

As the prevalence of antibiotic drug resistance increases, arguments for combination therapy are getting more traction. Thus, we tested the potential for synergy between CBD and different conventional drugs (clindamycin, ofloxacin, meropenem, tobramycin, methicillin, teicoplanin, and vancomycin) against MRSA. CBD demonstrated a promising MIC value at 1 µg/mL against MRSA strain USA300. The FIC_index_ results for the combination by the checkerboard method yielded indifferent effects against the tested strain yet no antagonism effect was observed (Table 2*).*

### 3.4. Minimum Bactericidal Concentration 

As CBD had antimicrobial activity against tested *S. aureus* MRSA USA300 and *S. aureus*, with minimum inhibitory concentration (MIC) 1 µg/mL, the MBC determination of CBD was obtained by spot plating the bacterial suspensions from the MIC plate on fresh agar plates and incubation at 37 °C for 24 h. The lowest concentration resulting in no viable bacterial colonies was reported as MBC. MBC was growing at concentrations of MIC, 2 × MIC, and 4 × MIC.

### 3.5. Optical Density Measurements

**Figure 3:**
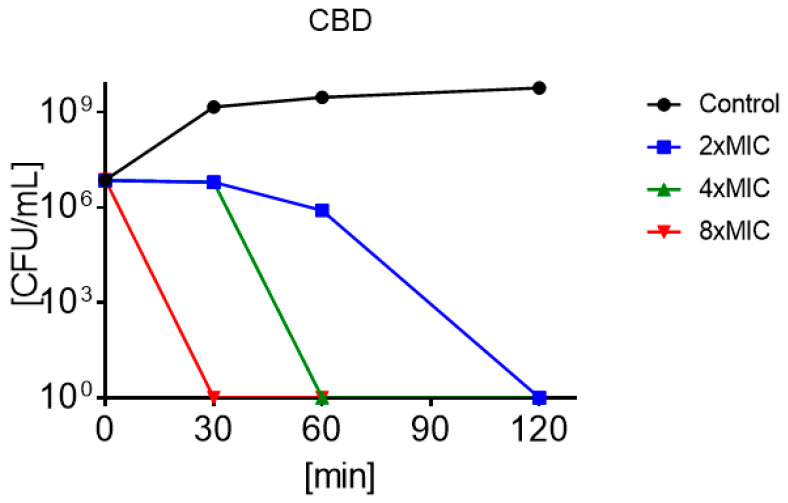
Time kill profiles of MRSA under treatment of 2 × minimum inhibitory concentration (MIC) (■), 4 × MIC (▲), and 8 × MIC (▼) of CBD and untreated control (●).

Optical measurements of the growth of the MRSA USA300 and *S. aureus* were monitored over time in the presence and absence of 2 × MIC, 4 × MIC, and 8 × MIC CBD. This comparative assessment of the antimicrobial activities of CBD over time indicated a significant inhibition of growth of both bacterial strains (*p* < 0.05). The graph depicted a flat growing curve due to a reduction of the optical density at the beginning of the lag phase (around 2 h), while the control continued in exponential growth. Further plating of aliquots after 22 h showed no colonies confirming the bactericidal effect of CBD (Figure 3). At 4 × MIC, CBD already decreased in growth following 30 min of incubation.

### 3.6. Cytotoxicity Test on HaCaT Cells

**Figure 4:**
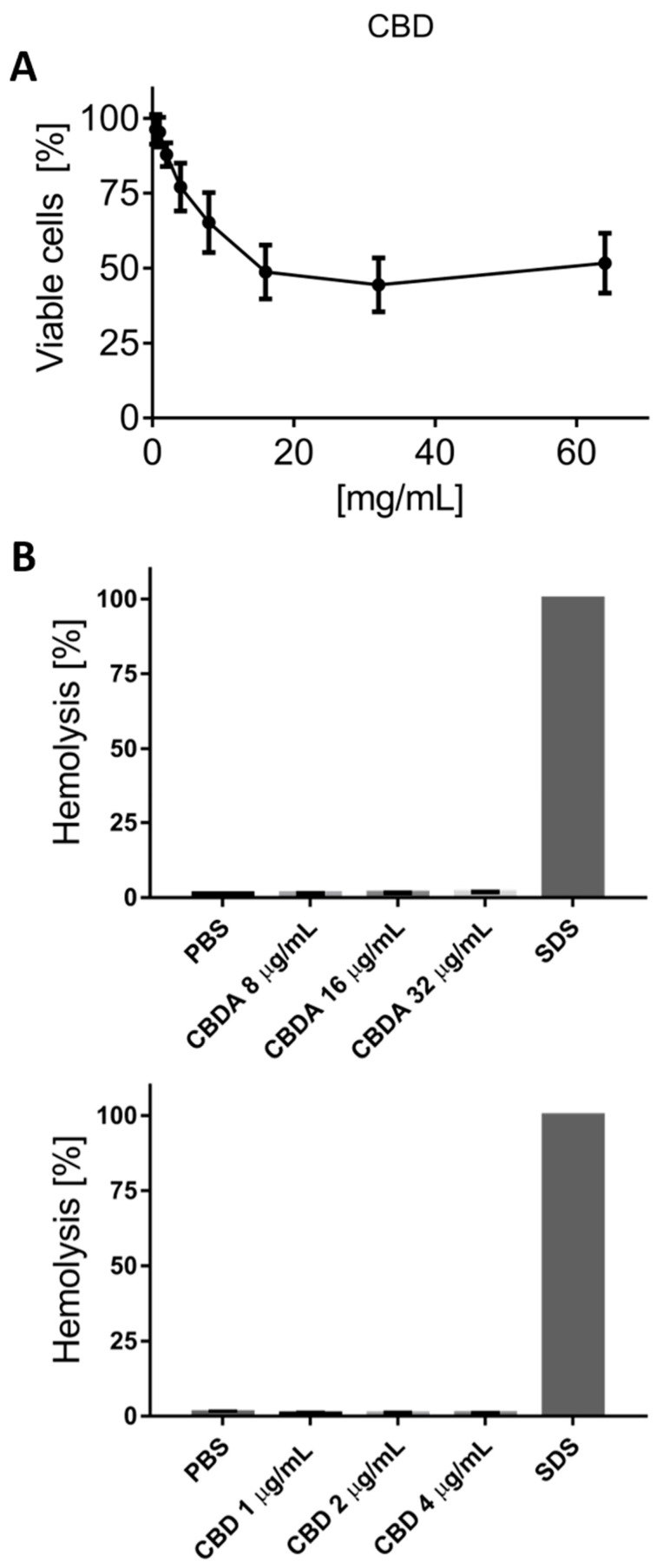
(**A**) The graph presents the percentage of viable cells and thus toxicity of the three MTS tests performed on human keratinocyte cells (HaCaT), with dose dependent killing up until 16 µg/mL of CBD, from where the toxicity levels out around 50% up to the highest tested concentration of 64 µg/mL of CBD. (**B**) CBDA and CBD demonstrate very minor hemolytic activity in concentration ranges of up to around 8 × MIC and 4 × MIC, respectively. SDS was used as a positive control lysing 100% of the red blood cells.

The safety of CBD toward human keratinocyte cells (HaCaT) was evaluated by MTS assay. Cells were treated with extracts at increasing concentrations between 0.5 and 64 µg/mL for 24 h, and the percentage of cell viability was analyzed (Figure 4). CBD at doses of 0.5, 1, 2, 4, and 8 µg/mL showed a dose dependent toxic effect, though at increased concentrations of CBD from 16 to 64 µg/mL. The HaCaT toxicity stabilized at around 50%. Hence, CBD presents little toxicity to human keratinocytes at concentrations of up to seven-fold higher than the compounds’ MIC values determined against MRSA USA300.

### 3.7. Hemolysis Test on Red Blood Cells

The safety against human red blood cells was evaluated with a hemolysis test. The hemolytic assay of CBD and CBDA on human erythrocytes did not show significant variations between the wide ranges of concentration (Figure 4*).* CBD in two-fold concentrations from 4 to 1 µg/mL presented values of ≤0.8%, while CBDA in concentrations ranging from 32 to 8 µg/mL pointed at values of ≤1.75%. Both results indicated a significant gap between MIC and hemolytic activity, an important screening parameter for further evaluation of the biological applications of CBD and CBDA [33]. 

## 4. Discussion

We were able to develop a method for the extraction, isolation, and quantification of the main cannabinoids, cannabidiolic acid (CBDA) and cannabidiol (CBD) from the fiber-type *Cannabis sativa* L. CBDA and CBD were obtained as a yellowish oil from the crude extract. The two compounds were efficiently extracted from dried hemp material by 96% ethanol. Methanol may also be used, though it tends to give a slightly lower extraction yield [34]. The content of CBDA and CBD in fiber-type *C. sativa* L. plants depends on several factors including the geographical location, soil, and environmental condition [35,36,37,38]. This can explain why we found an approximately three times higher amount of isolated CBDA in the hemp from Canna Therapeutics compared to the CBDA levels in the Cannabis from Møllerup Gods. Purification of these phytochemicals were sufficiently obtained using solid-phase extraction reverse phase C18 chromatography, with an increased gradient of methanol. Further purification by normal phase chromatography, as suggested by Appendino et al. [21], did not improve the purity of CBDA in our research. Isolates of CBDA and CBD demonstrated to be more stable when stored in DMSO than methanol likely due to a higher boiling point and less volatile properties of DMSO (data not shown). Thermal decomposition of CBDA was also avoided by storage at 4 °C and darkness for up to 10 days. Contrary, methylation of CBD was observed after 10 days of storage at 4 °C, thus −20 °C is preferred for long-term storage. 

All Gram-positive bacterial strains tested demonstrated high sensitivity towards CBD, with slightly lower effects by CBDA. A two- to four-fold decrease in the antibiotic effectivity of the polar analogue of CBD substantially suggests that the antibacterial pharmacophore falls on the resorcinol moiety of these cannabinoids and not the terpenoid and n-pentyl groups functioning as modulators for lipophilicity. The overlapping chemical features of the resorcinol moiety and simple phenols are striking. As phenols present exceptional bactericidal abilities features [39], it is tempting to draw parallels to our findings. However, at the same time, it is important to emphasize that the decarboxylation of the resorcinol moiety has a great biological repercussion, resulting in the decrease of hydrophilicity, and thus considerably reducing the membrane permeability [40] negatively impacting on its antimicrobial potential. Earlier studies have also demonstrated that a resorcinol molecule on its own did not exhibit any antimicrobial activity on *Staphylococcus* even at 256 µg/mL [21], clearly corroborating the importance of the other moieties in CBD as well in respect to antibacterial activity (Figure 1). The importance of the terpenoid is further supported by studies, demonstrating that olivetol only possesses a modest antimicrobial activity against Gram-positive bacteria ranging from 64 to 128 µg/mL [21].

It is also noteworthy that CBD and CBDA demonstrated no significant inhibitory effect on the tested Gram-negative strains *E. coli* and *P*. *aeruginosa*. These results might be because Gram-negative bacteria have lipopolysaccharides and proteins with very little phospholipid in the outer leaflet of the outer membrane—the decisive factor in the effective detergent resistance of this membrane. It is impermeable to macromolecules and allows only limited diffusion of hydrophobic molecules. In addition, the outer membrane of this bacteria is resistant to neutral and anionic detergents [41].

Due to this great antimicrobial potency of CBD against MRSA, the synergy with conventional antibiotics was tested. CBD was not able to revert the resistance pattern or demonstrate synergy with any of the conventional antibiotics tested. Thus, despite the hydrophobic potential of CBD, it does not seem like CBD disturbs the bacterial membrane enough to enhance the uptake of conventional drugs, as has been seen for many other novel membrane disturbing drugs [42,43]. On the other hand, it is important to emphasize that CBD did demonstrate additive effects with several last resort drugs and that the antibacterial mechanism of action of CBDA and CBD seems to not interfere with classical antibiotics.

Time-killing studies of CBD against MRSA USA300 and *S. aureus* (ATCC 25923) have shown that the compound is highly bactericidal and with a very rapid killing effect (Figure 3). This has resemblance with saponins, a natural non-ionic detergent with cell membrane-permeabilizing properties [44]. Contrary, selectivity against Gram-positive over Gram-negative bacterial can argue that the peptidoglycan layer is the main target. This is further corroborated by the very low hemolytic activity (Figure 4), and studies are currently being conducted to better characterize this mode of action. The toxicity reported against the HaCaT cells are rather significant and would be concerning if reported alone. However, the fact that CBD experimentally has been tested in mammalian and human trials at concentrations of up to 3500 mg/day [45,46,47,48] will argue that epithelial cell toxicity is less of an issue. Oral admission of as much as 6000 mg/day has also documented no adverse side-effects [49], arguing in favor for the safety of CBD at concentrations that are 1000 times higher than MIC.

## 5. Conclusions

CBD displays a potent MIC against clinically relevant Gram-positive bacteria. Though it does not possess synergy with any of the tested conventional antibiotics, CBD may still be an interesting addition to current antimicrobial regiments, particularly due to its low toxicity profile and rapid bacterial killing.

## Figures and Tables

**Table 1:** Minimal inhibitory concentration of CBDA and CBD along with key antibiotics towards a panel of Gram-positive and Gram-negative species. CBDA is slightly less potent than CBD against Gram-positive strains, while both are inactive against Gram-negatives strains.

Minimal Inhibitory Concentration [MIC µg/mL]						
	*Staphylococcus Aureus*		*Staphylococcus Epidermidis*		*Escherichia Coli*	*Pseudomonas Aeruginosa*
	(ATCC 25923)	MRSA (USA300)	(CA#71)	(ATCC 51625)	(ATCC 25922)	(PA01)
CBDA	2	4	4	4	>64	>64
CBD	1	1	2	2	>64	>64
Clindamycin	>128	128	>128	0.06	8	>128
Tobramycin	0.25	1	0.06	1	2	0.12
Meropenem	0.06	16	0.12	2	0.06	0.5
Ofloxacin	0.5	64	0.25	1	0.06	1

**Table 2:** Synergy test on methicillin-resistant *Staphylococcus aureus* (MRSA) USA300, two-fold dilution series of both antibiotics and CBD, 128–0.5 µg/mL and 16–0.25 µg/mL, respectively. FIC_index_ ≤ 0.5 is considered synergy; FIC_index_ between 0.5 and 1 is considered additive; FIC_index_ between 1 and 4 is considered indifferent; and FIC_index_ ≥ 4 is considered antagonist.

Drug Combination	FIC_index_
CBD + Clindamycin	1.03
CBD + Vancomycin	1.13
CBD + Tobramycin	1.50
CBD + Teicoplanin	1.13
CBD + Ofloxacin	1.50
CBD + Methicillin	1.25
CBD + Meropenem	1.13

FIC: fractional inhibitory concentration.

## Supplementary Materials

The following are available online at https://www.mdpi.com/2218-273X/10/6/900/s1, Figure S1: CBDA and CBD standard curves, Figure S2: CBD chromatogram and retention timetable, Figure S3: CBDA chromatogram and retention timetable.
