# Supplementary material for: Isolation, Purification, and Antimicrobial Characterization of Cannabidiolic Acid and Cannabidiol from Cannabis sativa L
Source: Biomolecules. 2020 Jun 12;10(6):900. doi: 10.3390/biom10060900 (PMC7355595; doi:10.3390/biom10060900)
Supplement: Supplementary file 1 [file biomolecules-10-00900-s001.pdf]

biomolecules

MDPI

Isolation, Purification, and Antimicrobial Characterization of Cannabidiolic Acid and Cannabidiol from *Cannabis sativa* L.

Laura Daniela Martinenghi, Rie Jønsson, Torben Lund and Håvard Jenssen *

Department of Science and Environment, Roskilde University, 4000 Roskilde, Denmark; laumarti80@yahoo.es (L.D.M.); riejonsson@gmail.com (R.J.); tlund@ruc.dk (T.L.)

* Correspondence: jenssen@ruc.dk; Tel.: +45 4674 2877

Supplementary data

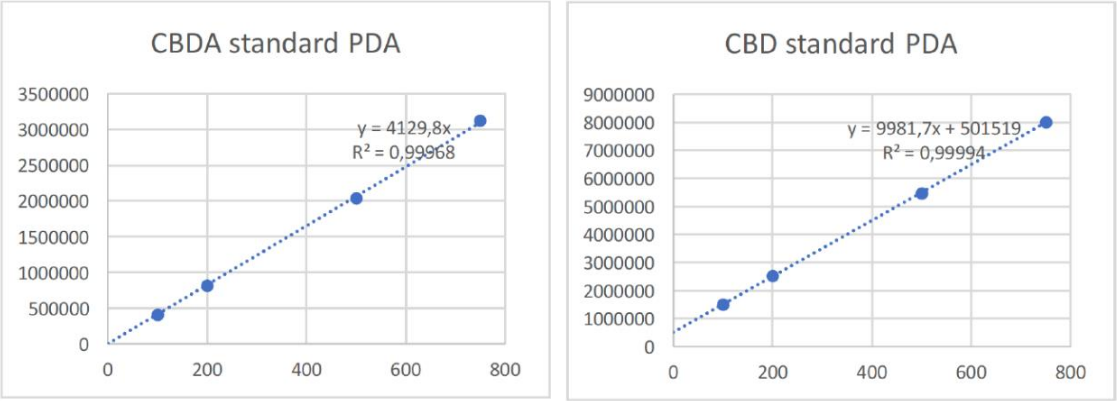

Figure S1. CBDA and CBD standard curves. Plotted at concentrations 100, 200, 500, and 750 µg/mL. On the y-axis the integration numbers corresponding to the area under the peak and on the *x*-axis the concentration of the compounds. Concentration of the samples were obtained by either using the graph or the linear equation.

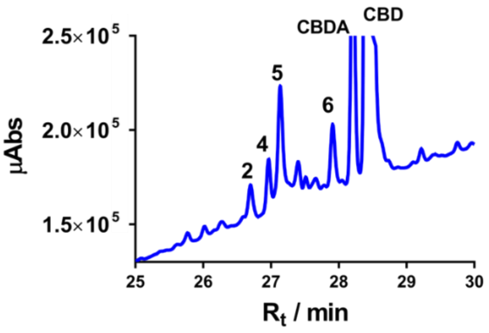

Compound	Rt/min	%Area	λmax/nm	*m*/*z*
2	26.70	0.8	270	331
4	26.96	0.8	272	?
5	27.14	2.2	277	345
6	27.91	1.2	272	?
CBDA	28.21	5.7	307	359
CBD	28.43	**89.7**	274	315

Figure S2. CBD chromatogram and retention-time table. CBD sample purified. The LC-UV/vis chromatogram is plotted in λmax mode between 225-800 nm. The layout is a zoomed version of Figure 2C from the main manuscript, to better illustrate the small peaks with contaminates. Integration of chromatogram are found in the table to the right.

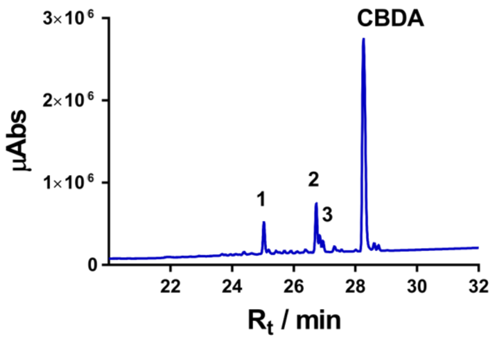

Compound	Rt/min	%Area	λmax/nm	*m*/*z*
**1**	24.38	3.1	343	343
**2**	26.73	9.8	307	331
**3**	26.95	2.3	300	375
**CBDA**	28.26	**84.9**	**307**	**359**

Figure S3. CBDA chromatogram and retention-time table. CBDA sample purified. The LC-UV/vis chromatogram is plotted in λmax mode between 225-800 nm. The chromatogram is matching CBDA Figure 2C from the main manuscript. Integration of chromatogram are found in the table to the right.
